# Word Reading Fluency: Role of Genome-Wide Single-Nucleotide Polymorphisms in Developmental Stability and Correlations With Print Exposure

**DOI:** 10.1111/cdev.12207

**Published:** 2014-01-06

**Authors:** Nicole Harlaar, Philip S. Dale, Maciej Trzaskowski, Robert Plomin

**Affiliations:** University of Colorado Boulder; University of New Mexico; King’s College London; King’s College London

## Abstract

The genetic effects on individual differences in reading development were examined using genome-wide complex trait analysis (GCTA) in a twin sample. In unrelated individuals (one twin per pair, *n* = 2,942), the GCTA-based heritability of reading fluency was ~20%-29% at ages 7 and 12. GCTA bivariate results showed that the phenotypic stability of reading fluency from 7 to 12 years (*r* = 0.69) is largely driven by genetic stability (genetic *r* = 0.69). Genetic effects on print exposure at age 12 were moderate (~26%) and correlated with those influencing reading fluency at 12 (genetic *r* = 0.89), indicative of a gene–environment correlation. These findings were largely consistent with quantitative genetic twin analyses that used both twins in each pair (*n* = 1,066-1,409).

One of the expectations of most education systems is that every child will be able to read fluently, effortlessly, and independently. This is not an easy task: “Building the reading brain” requires effort and incremental skill development throughout the school years (Nevills & Wolfe, 2009). Decades of research have improved our understanding of the cognitive skills that underpin good reading, as well as the teaching practices that may facilitate the development of these skills. Nonetheless, there are strikingly large and stable individual differences in reading ability and achievement, even among children of the same age and in the same classroom. Literacy environments at home and at school, encompassing factors such as shared book reading, literacy-rich classrooms, and the quality and quantity of reading instruction in schools, only partially account for these individual differences and their stability. For a more complete understanding of individual differences in reading development, it is clear that genetic differences among individuals must also be considered.

The notion that individual differences in reading are partly due to genetic influences is supported by over two decades of twin research (Olson, Keenan, Byrne, & Samuelsson, 2013). Significant heritability estimates have been reported for individual differences in diverse reading skills (e.g., Byrne et al., 2013; Keenan, Betjemann, Wadsworth, DeFries, & Olson, 2006; Taylor & Schatschneider, 2010) and for the risk of developing reading difficulties (e.g., Astrom, Wadsworth, Olson, Willcutt, & DeFries, 2011; Harlaar, Spinath, Dale, & Plomin, 2005). Furthermore, longitudinal twin studies have shown that genetic factors contribute importantly to individual differences in the rank order stability of reading performance (e.g., Betjemann et al., 2008; Harlaar, Dale, & Plomin, 2007a) and to the persistence of reading disabilities (Astrom et al., 2011). Finally, genetic factors partly account for individual differences in the rate of children’s reading growth (e.g., Christopher et al., 2013).

A second finding from twin studies is that aspects of the child’s literacy environment, such as their cumulative reading experience, or *print exposure*, also partly reflect genetic variation, and these genetic influences are associated with individual differences in reading skill (e.g., Harlaar, Deater-Deckard, Thompson, DeThorne, & Petrill, 2011; Martin et al., 2009). This is an example of an *active gene*-*environment correlation*, where an individual’s genetically influenced traits are associated with the environmental niches selected by the individual. That is, a child with a higher proportion of genes that positively influence reading development may be more likely to seek out opportunities to read compared to a child who is at greater genetic risk for reading disabilities. Many putative environmental factors are heritable (e.g., Kendler & Baker, 2007), indicating that gene–environment correlations are likely to be widespread in development.

One index of the degree to which genetic factors account for the phenotypic stability of reading or the association with reading-related experiences is the *genetic correlation*, which is the correlation of the genotypic effects for two measures. Genetic correlations may be estimated between reading measures obtained at different times (in a longitudinal study), or between measures of reading and an environmental factor (in a study of gene–environment correlation). Longitudinal twin studies of reading skill in school-age children have reported genetic correlations of around 0.50-1.00 across ages, suggesting that the same genetic influences largely account for variation in both early and later reading skill (e.g., Betjemann et al., 2008; Harlaar et al., 2007a). There have been relatively few studies that have specifically estimated the correlation between genes and environments in reading development. However, using the Twins Early Development Study (TEDS; Haworth, Davis, & Plomin, 2013), we have previously found that individual differences in print exposure at age 10 as assessed by the Author Recognition Test (ART) are predicted by word reading fluency at age 7, assessed by the Test of Word Reading Efficiency (TOWRE; Harlaar, Dale, & Plomin, 2007b). Genetic factors accounted for around 10% of individual differences in ART scores, and this heritability was almost completely accounted for by genetic factors that also contributed to variation in 7-year TOWRE scores. This finding is suggestive of a genetically influenced path that prospectively links risk for early reading failure with later print exposure.

## Genome-Wide Complex Trait Analysis: A New Way to Examine the Effects of Genetic Variation on Complex Traits Using DNA Alone

Although the twin design continues to be invaluable for investigating the etiology of complex traits such as reading skill (Haworth & Plomin, 2010), the era of genome-wide genotyping now provides tools to examine the genetic etiology of reading development using DNA alone. Genome-wide genotyping is made possible by DNA arrays that assay many single-nucleotide polymorphisms (SNPs) simultaneously. The most well-known application of genome-wide genotyping is the genome-wide association (GWA) study, in which phenotypic variation is associated with differences in allele frequency at each SNP. The current generation of DNA arrays allows researchers to measure up to around 4.5 million SNPs for each individual. GWA studies also often include imputed SNPs, unobserved genetic variants that are inferred from reference panels that include detailed information on a much larger number of markers. An association between an SNP and the phenotype may arise if the SNP itself is the functional genetic variant, or if the SNP is very close to it (i.e., in *linkage disequilibrium* with the true causal allele). Due to the large number of SNPs tested, a threshold of *p* ≤ .05 × 10^−8^ is typically used to control the rate of Type I error.

GWA studies have been successful in identifying SNPs for some complex quantitative traits, such as height and weight (Grigorenko & Dozier, 2013; Visscher, Brown, McCarthy, & Yang, 2012). However, the results from GWA studies of reading and other educationally relevant phenotypes have so far been limited (Plomin, 2013). In a study using the TEDS sample at age 7 (Meaburn, Harlaar, Craig, Schalkwyk, & Plomin, 2008), an initial discovery stage based on “pools” of DNA from low- and high-reading-ability groups (*n* = 750 in each group) identified 10 nominally significant SNPs that replicated in a second TEDS subsample of individually genotyped individuals. However, none of the SNP associations accounted for more than 0.5% of the variance in reading ability. More recently, a GWA metaanalysis of reading measures was conducted on two population-based samples, consisting of British children (*n* = 5,472) and primarily adolescent Australians (*n* = 1,177; Luciano et al., 2013). The most robust result, an SNP in the DAZ-associated protein 1 (*DA-ZAP1*) gene, was nominally associated with two reading phenotypes yet did not account for more than 0.4% of the variance in reading. Finally, an even larger GWA study (*n* = 126,599) on educational attainment identified and replicated three SNPs that were significantly associated with years of education (Rietveld et al., 2013). The SNP with the strongest effect explained only 0.02% of the variance, corresponding to a difference of around 1 month of schooling per allele. The results from these studies indicate that the average effect size of individual genetic variants for educationally relevant traits is extremely small, even when samples of hundreds of thousands of individuals are available.

Notwithstanding these sobering conclusions, it has become clear that the data from genome-wide DNA arrays can be leveraged in other ways. Notably, it is possible to derive genetic similarities at measured SNPs among classically unrelated individuals and then use those similarities to estimate the amount of variability explained by all SNPs together (Visscher, Yang, & Goddard, 2010; Yang, Lee, Goddard, & Visscher, 2011). We will refer to this method as genome-wide complex trait analysis (GCTA), although other names and analytic variants have been proposed (Plomin & Simpson, 2013; Zaitlen & Kraft, 2012). GCTA is analogous to the standard twin method, which compares monozygotic (MZ) twins, who are genetically identical, with dizygotic (DZ) twins, who share 50% of their segregating genes, on average. Rather than using twins, however, GCTA examines distant genetic relatedness among pairs of unrelated individuals who have been genotyped on a DNA array. Although chance DNA similarity between pairs of unrelated individuals is tiny (< 2%), overall genetic influence, or *GCTA-based heritability*, can be estimated from the extent to which this random DNA similarity predicts their phenotypic similarity. Thus, the GCTA-based heritability may be defined as the proportion of variance due to the additive effects of common SNPs represented across a DNA array.

The first published GCTA studies showed that SNPs explained around 45% of the phenotypic variance in height (Yang et al., 2010). This GCTA-based heritability can be contrasted with the results from a meta-analysis of 46 GWA studies of height (*n* = 183,727), which identified 180 SNPs that collectively accounted for around 10% of the phenotypic variance (Lango et al., 2010). These findings indicate that GCTA-based heritability includes the additive effects of many common SNPs that individually are too small to be statistically significant in a GWA study. However, the GCTA-based heritability estimate is substantially lower than the results from twin studies, which have reported heritabilities of around 70%-90% for height (e.g., Silventoinen et al., 2003).

The discrepancy between the GCTA-based heritability and twin study estimates of heritability is sometimes referred to as the *missing heritability problem*, and can be attributed to incomplete linkage disequilibrium between the SNPs on DNA arrays and other causal genetic variants. The DNA arrays used in GWA studies are not unbiased. They assay only a fraction of all SNPs in the genome, principally “common” SNPs that have a minor allele frequency of between 5% and 50% (i.e., between 5% and 50% of the sampled population will have the less common allele of the SNP). Common SNPs are preferentially selected for DNA arrays because they are more informative in terms of providing coverage of the entire genome. A corollary of this bias is that if some of the genetic variants that contribute to variance in a trait are relatively rare, or if they are poorly tagged by the SNPs on the array (i.e., they are not in linkage disequilibrium with genotyped SNPs), the GCTA-based heritability will fall short of heritability estimates from twin studies (Plomin, 2013).

Since Yang et al.’s (2010) original publication, GCTA has been applied to a range of traits and diseases, including developmentally relevant phenotypes such as cognitive abilities (e.g., Deary et al., 2012; Plomin, Haworth, Meaburn, Price, & Davis, 2013), substance use (Vrieze, McGue, Miller, Hicks, & Iacono, 2013), and stressful life events (Power et al., 2013). The findings from these studies have generally mirrored the pattern observed for height. That is, GCTA heritabilities are often considerably lower than those estimated in twin studies, but larger than the combined effects of the top SNPs identified in GWA studies, where available (although there are exceptions; e.g., Trzaskowski, Eley, et al., 2013; reported negligible GCTA heritability for anxiety).

The value of GCTA has recently been greatly increased by extending it beyond the univariate analysis of the variance of a single trait to the bivariate analysis of the covariance between two traits (Lee, Yang, Goddard, Visscher, & Wray, 2012). This adaptation allows us to estimate a GCTA-based genetic correlation, reflecting the extent to which common SNPs contribute to the correlation between two phenotypes. The first application of this approach was to longitudinal IQ data (Deary et al., 2012), which showed that the phenotypic stability of IQ from age 11 to age 70 (phenotypic *r* = 0.63) is largely driven by genetic stability (genetic *r* = 0.62). We obtained similar findings in a bivariate GCTA of general cognitive ability (*g*) in childhood from ages 7 to 12 using the TEDS sample (Trzaskowski, Yang, Visscher, & Plomin, 2013). In this study, we also took advantage of the fact that we had a twin sample to compare twin study and GCTA estimates. The GCTA-based genetic correlation from ages 7 to 12 was 0.73, highly similar to the genetic correlation of 0.75 estimated from an analysis using all twins. Thus, the GCTA confirmed the results of twin studies indicating strong genetic stability for *g*.

## The Current Study

Twin studies have provided robust evidence that individual differences in reading partly reflect stable genetic differences among children, but the results of GWA efforts to identify specific SNPs that may account for the heritability of these individual differences have so far been limited. Since the publication of our original GWA study of reading skill (Meaburn et al., 2008), our molecular genetic efforts have continued. Individual genotypes are now available on almost 2 million measured and imputed SNPs for a subsample of TEDS. In this study, we examine the extent to which bivariate GCTA confirms predictions arising from quantitative genetic twin analyses. In addition, the availability of twins permits the direct comparison of GCTA-based heritabilities and genetic correlations with twin-based heritabilities and genetic correlations, as we have done in previous GCTA studies (Plomin, Haworth, et al., 2013; Trzaskowski, Davis, et al., 2013; Trzaskowski, Yang, et al., 2013).

We focus specifically on the efficiency and accuracy (i.e., fluency) of word reading skills, as assessed by the TOWRE at ages 7 and 12. Attaining word reading fluency is a key goal for the early school years: When students are able to read most of the words in text quickly and accurately, they can concentrate on the meaning of the text and they are more likely to understand and remember what they read. Our goals were twofold. First, we sought to examine the extent to which common SNPs genotyped on or tagged by current DNA arrays account for variance in TOWRE scores at ages 7 and 12, as well as the extent to which these SNPs correlate across this 5-year span. In a previous study, we found that a composite of four reading measures at age 12 showed significant GCTA-based heritability (Trzaskowski, Davis, et al., 2013). Given this finding, we hypothesized that the GCTA-based heritability estimate for TOWRE scores would be significantly greater than zero but lower than the twin-based heritability estimates, on the assumption that DNA arrays capture some, but not all, traitrelated genetic variants. In addition, given evidence for the genetic stability of reading from twin studies, we predicted that common SNPs that account for genetic variance in TOWRE scores at age 7 would correlate positively with SNPs that account for genetic variance in TOWRE scores at age 12.

Second, we examined the extent to which SNPs account for variance in print exposure at age 12. Although our previous study (Harlaar et al., 2007b) showed that individual differences in ART scores at age 10 were only modestly heritable, we anticipated that genetic influences on print exposure increase from ages 10 to 12 due to gene–environment correlations that cause children to seek out reading environments consistent with their genetic propensities (see also Martin et al., 2009, who estimated the heritability of ART scores in primarily adolescent Australian twins to be 67%). Accordingly, we predicted that common SNPs, in aggregate, would partly account for the variance in ART scores, and that this SNP-based variance would correlate positively with SNPs that account for variance in TOWRE scores.

## Method

### Participants

The sample was drawn from TEDS, a prospective population-based cohort of over 11,000 twin pairs born in England and Wales between January 1994 and December 1996 (Haworth et al., 2013; Kovas, Haworth, Dale, & Plomin, 2007). To minimize the effects of population stratification, our analyses were restricted to White families only (~93% of the TEDS sample, which is similar to the proportion of White families in England and Wales for this generation of children; Haworth et al., 2013). In addition, we excluded children with a history of severe medical or psychiatric problems. Genome-wide genotyping was completed in 2010 for one randomly selected child in each of 3,665 families using DNA extracted from buccal cheek swabs. The genotyping was performed on Affymetrix GeneChip 6.0 SNP arrays as part of the Wellcome Trust Case Control Consortium 2 (WTCCC2; described in Trzaskowski, Eley, et al., 2013). Of the individuals genotyped, a total of 513 samples were excluded because they did not meet quality control criteria (including low call rate, unusual heterozygosity, unusual hybridization intensity, atypical population ancestry, sample duplication or relatedness to other sample members, and gender mismatches). For this study, we also excluded 22 individuals who did not have word reading fluency or print exposure data at either age 7 or age 12, yielding a total of 3,130 individuals (54.2% females) for our analyses.

A key assumption of GCTA is that pair-by-pair genetic similarity is random. For this reason, we used one individual per twin pair and we excluded pairs of individuals who were genetically related more than .025 (comparable to fourth cousins). For the twin analyses, we used both the twin in the GCTA and their cotwin. Twin zygosity was assigned on the basis of physical appearance or DNA similarity (Kovas et al., 2007). This study included 1,202 MZ twin pairs (527 males, 675 females) and 1,928 DZ twin pairs (447 males, 549 females, 932 opposite sex). DZ same-sex and opposite-sex pairs were combined to increase power and because previous twin analyses of these data show no evidence of qualitative or quantitative sex differences in sex-limitation models (Kovas et al., 2007). Ethical approval was provided by King’s College London’s Ethics Committee. Informed consent was obtained from parents for each part of the study prior to data collection.

### Measures

#### Word Reading Fluency

Twins in each pair were assessed separately on the TOWRE (Torgesen, Wagner, & Rashotte, 1999) by telephone at ages 7 and 12. The TOWRE has two timed (45 s) subtests, phonological decoding efficiency (PDE), which requires reading decodable pseudowords (e.g., *tegwop*), and sight word reading (SWE), which requires reading single real words. Test stimuli were mailed in sealed envelopes to twins in advance of the telephone testing session, with instructions that the envelopes should not be opened prior to the test session.

The PDE and SWE subtests were substantially correlated both phenotypically (0.83 at age 7; 0.74 at age 12) and genetically (e.g., genetic correlations of 0.89 at age 7; 0.92 at age 12; further details available from the first author). The high genetic coefficients imply that the key components of word reading fluency measured by these subtests—reading real words quickly and being able to decode irregular words—are influenced almost completely by the same genetic factors. Given this high degree of overlap, all subsequent analyses were conducted on overall TOWRE scores, calculated as the mean of PDE and SWE scores at each age.

#### Print Exposure

We used an online adaptation of the ART (Stanovich & West, 1989) to assess print exposure at age 12. This test follows a quick-probe logic in which participants are given a list of author names intermixed with a set of foils, and are asked to indicate which items they recognize as the names of real authors. Pilot testing revealed that many authors on the original list used by Stanovich and West (1989) were not familiar to our sample. Accordingly, we developed a list of 21 authors using online lists of classic and more recently popular books aimed at children between 10 and 14 years. We included 21 “foils,” matched in length and gender to the real author names that we created using an online random name generator. Children were asked to identify authors who wrote books for children by checking either a “Yes” or “No” box against each name.

In our online version of the ART, instructions were presented in both written and audio forms to reduce reading load. Scoring was determined by taking the proportion of author names checked and subtracting the proportion of foils checked. Test-retest reliability of the ART across 2 weeks in a subsample of 37 twin pairs in TEDS was 0.96. As noted in the Introduction, the ART was also administered at age 10. These data were not analyzed in this study because the available sample with genome-wide genotyping was relatively small (< 1,000 individuals), which would provide substantially reduced power for bivariate GCTA.

#### Preprocessing

To create composite scores for the TOWRE and ART measures, age- and sex-standardized residuals were derived for each scale. Outliers above or below 3 *SD* from the mean were excluded and scores were normalized by transforming the ranked data to the quantiles of a standard normal distribution using the van der Waerden (1952) transformation. The *R* statistical environment (R Core Team, 2013) was used for these preprocessing steps.

### Genotyping

Our GCTA was based on a total of 1,724,317 autosomal SNPs, which included around 700,000 genotyped SNPs and 1 million imputed SNPs. Briefly, raw image data from the AffymetrixGeneChip 6.0 SNP genotyping arrays were normalized and preprocessed at the Wellcome Trust Sanger Institute (Hinxton, UK). Genotyped SNPs were retained on the basis of minor allele frequency (> .01), genotype call rate (> .80), Hardy-Weinberg equilibrium (> 10^−20^), and plate effect *p* value (> 10^−6^). Imputed SNPs were generated in IMPUTE v.2 (Howie, Donnelley, & Marchini, 2009) using reference panels from HapMap 2 and 3 and the WTCCC controls. Only imputed SNPs with an information score ≥ 98 were retained for analysis. Further details of the genotyping and imputation procedures are provided in Trzaskowski, Eley, et al. (2013). To control for ancestral stratification, we performed principal component analysis using the EIGENSOFT package (Price et al., 2006). We identified eight axes (*p* < .05) using the Tracy–Widom test (Patterson, Price, & Reich, 2006) that we subsequently included as covariates in our GCTA analyses.

### Statistical Analyses

#### GCTA

As described in the Introduction, GCTA estimates the extent to which genetic variance captured by common genome-wide SNPs on a DNA array account for the variance of quantitative traits in unrelated individuals (Visscher et al., 2010; Yang et al., 2011). More specifically, GCTA evaluates the joint effect of overall genetic similarity pair by pair for all SNPs considered simultaneously as a random effect, and then estimates the variance in the phenotype attributable to this random effect. The basic analysis has two steps. First, a genetic relationship matrix is estimated based on the genetic similarity between all pairs of subjects using all genetic markers genotyped on the DNA array. In the second step, the genetic relationship matrix is compared to a matrix of pairwise phenotypic similarity using a restricted maximum likelihood random-effects mixed linear model.

GCTA yields an estimate of the additive variance in the trait accounted for by the SNPs assessed on a DNA array. It also estimates a residual component, reflecting variance due to random noise, unmeasured environmental influences, and unmeasured genetic variants that are not in linkage disequilibrium with genotyped SNPs (including variants with nonadditive effects). Bivariate GCTA extends the univariate GCTA model by relating the pairwise genetic similarity matrix to a phenotypic covariance matrix between the two traits of interest, allowing for correlated residuals (Lee, Yang, et al., 2012). Using bivariate GCTA, we estimated the proportion of variance due to the aggregate effects of SNPs on each variable (GCTA-based heritability), and the extent to which the aggregate effects correlate across variables (GCTA-based genetic correlation). The same parameters were estimated for the residual effects. Correlations between the residual components were estimated using formulae detailed in Trzaskowski, Yang, et al. (2013).

#### Twin Modeling

Our twin analyses used both members of a twin pair (i.e., the twin selected for GCTA plus their cotwin). Briefly, the phenotypic variance within each variable and the covariance between variables is apportioned among three components: *additive genetic* (A) influences, reflecting variation in genotypes transmitted from parents to offspring; *shared environmental* (C) influences, reflecting nongenetic influences that affect all persons within a family (e.g., family socioeconomic status); and *nonshared environmental* (E) influences, reflecting variation in environment influences that cause individual family members to differ from one another (e.g., differential educational experiences). These variance components may be estimated from the comparison of phenotypic similarity in MZ and DZ twins (Plomin, DeFries, Knopik, & Neiderhiser, 2013). Based on the assumptions that trait-relevant shared environmental influences affect MZ and DZ twins equally and trait-relevant nonshared environmental influences do not contribute to similarity in either type of twin, the shared and nonshared environmental components of variance are considered invariant across zygosity. In contrast, genetic factors do vary as a function of zygosity: MZ twins are genetically identical, whereas DZ twins share 50% of their segregating genes, on average.

Our twin analyses used a Cholesky decomposition model (Gillespie & Martin, 2005). For each variable, we estimated the proportion of variance due to A, C, and E (denoted as a^2^, c^2^, and e^2^). We also computed the genetic (*r*_g_), shared environment (*r*_c_), and nonshared environment correlations (*r*_e_) between variables. These analyses were run in OpenMx (Boker et al., 2011) using full-information maximum likelihood.

## Results

Pearson correlations (with 95% confidence intervals) among measures are shown in Table 1. Two findings are of note. First, TOWRE scores at ages 7 and 12 were substantially correlated phenotypically (*r* = 0.69), indicating that word reading fluency is highly stable across this 5-year period. Second, ART scores were significantly correlated with TOWRE scores at both age 7 (*r* = 0.46) and age 12 (*r* = 0.43), indicating that children who read words fluently showed higher levels of print exposure compared with children with less fluent reading skills.

### To What Extent Do Common Genetic Variants Account for Variance in Word Reading Fluency Skills and the Stability of Individual Differences in Word Reading Fluency Skills From Ages 7 to 12?

**Figure 1:**
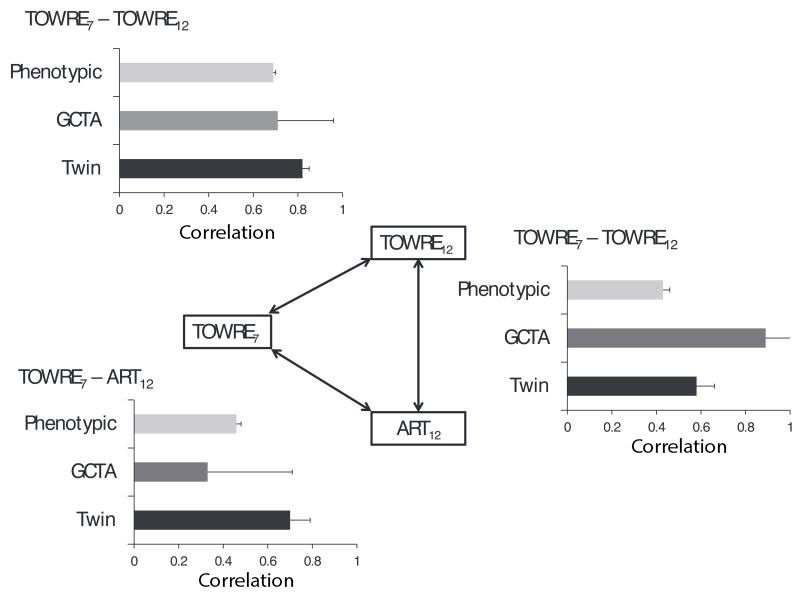
Summary of phenotypic and genetic (GCTA SNP-based estimates and twin-based estimates) correlations between TOWRE scores at ages 7 and 12 and ART scores at age 12, with standard errors. All standard error bars are constrained to no more than the maximum correlation value of 1.0. GCTA = genome-wide complex trait analysis; SNP = single-nucleotide polymorphisms; TOWRE = Test of Word Reading Efficiency.

Given that TOWRE scores at ages 7 and 12 showed substantial developmental stability, our first question of interest was whether common genetic variants account for variance in TOWRE scores and the observed stability from ages 7 to 12. Table 2 shows the parameter estimates from the GCTA and twin analyses of TOWRE scores at ages 7 and 12. Approximately 21% of the variance in TOWRE scores at age 7 was due to the aggregate effect of genotyped and imputed SNPs; this estimate was not significantly different from zero. The GCTA-based heritability of TOWRE scores at age 12, estimated as 29%, was significant. The corresponding twin heritability estimates were 74% and 68%, respectively. The GCTA-estimated genetic correlation was 0.71, similar to the twin study estimate of the genetic correlation (0.82); both estimates were significant. Thus, there is substantial genetic stability in variance in word reading fluency from ages 7 to 12, mirroring the phenotypic stability and twin results. The GCTA and twin-based estimates of the genetic correlations, along with the phenotypic correlation between TOWRE scores at ages 7 and 12, are summarized in Figure 1 (upper left corner).

Residual and environmental effects are also reported in Table 2. The residual effects estimated from GCTA were substantial and significant. The residual correlation (0.69) indicates that the stability of TOWRE scores from ages 7 to 12 is partly due to systematic factors that are independent of the additive effects of common SNPs. These factors could include genetic influences as well as environmental factors. In the twin analyses, shared environmental effects were small and generally nonsignificant (accounting for 7%-9% of the variance in TOWRE scores; shared environmental correlation = 0.39), whereas nonshared environmental effects were somewhat larger and significantly greater than zero (accounting for 17%-25% of the variance in TOWRE scores; nonshared environmental correlation = 0.36).

### To What Extent Do Common Genetic Variants Account for Variance in Print Exposure and the Correlation Between Print Exposure and Word Reading Fluency Skills?

Our second major question of interest was whether common genetic variants also account for variance in print exposure and the phenotypic correlations between print exposure and word reading fluency. We first ran GCTA and twin analyses on TOWRE and ART scores at age 12 to examine the concurrent association of word reading fluency and print exposure. Parameter estimates from these analyses are reported in Table 3. The GCTA-based heritability of individual differences in TOWRE scores at age 12 was 22%, whereas the corresponding twin-based heritability was 70%. The GCTA-based heritability of individual differences in ART scores at age 12 was 26%, whereas the corresponding twin-based heritability was lower, at 39%. As well as showing a significant genetic contribution to individual differences in ART scores, there is some evidence that these genetic effects are partly associated with TOWRE scores. The GCTA bivariate analysis yielded a significant GCTA-based correlation of 0.89. This correlation is not significantly different from the twin-based genetic correlation of 0.58, as indicated by the overlapping confidence intervals. The genetic correlations from both the GCTA and twin analyses, along with the phenotypic correlation between 12-year TOWRE and ART scores, are summarized in Figure 1 (center right).

We also examined the extent to which genetic influences on early word reading fluency predict later print exposure; parameter estimates from these analyses are reported in Table 4. Genetic effect sizes from the GCTA were small to medium in magnitude, and not significantly different from zero. Briefly, the GCTA-based heritability estimates for individual differences in 7-year TOWRE and 12-year ART scores were 28% and 21%, respectively; the GCTA-based correlation between these phenotypes was 0.33. The results from the twin analysis mirrored those reported in Table 3 for the concurrent analysis of 12-year TOWRE and ART scores: Genetic influences accounted for a large proportion of the variance in 7-year TOWRE scores (73%) and a moderate proportion of the variance in 12-year ART scores (27%), and these genetic effects were substantially correlated (genetic *r* = 0.70). The genetic correlations from both the GCTA and twin analyses, along with the phenotypic correlation between 7-year TOWRE and 12-year ART scores, are summarized in Figure 1 (lower left corner).

The GCTA- and twin-based analyses of the concurrent association between 12-year TOWRE and ART scores (Table 3) and of the prospective association between 7-year TOWRE scores and 12-year ART scores (Table 4) yielded largely similar estimates for the residual and environmental components of variance. Briefly, residual variance estimated from GCTA was substantial and significant for both TOWRE and ART scores (72%-79%), and the correlation between these residual effects was significantly greater than zero (*r* = 0.27 between 12-year TOWRE and 12-year ART scores; *r* = 0.50 between 7-year TOWRE and 12-year ART scores). In the twin analyses, environmental influences on 7- and 12-year TOWRE scores were primarily nonshared rather than shared (23% vs. 7% for 12-year TOWRE scores; 18% vs. 9% for 7-year TOWRE scores), whereas environmental influences on 12-year ART scores were due to both shared and nonshared environmental factors (32% vs. 29% in Table 3; 42% vs. 32% in Table 4). Shared environmental correlations between TOWRE and ART scores were substantial and significant (0.66 in Table 3; 0.64 in Table 4), but the nonshared environmental correlations were small and only significant for the analysis of 7-year TOWRE and 12-year ART scores, indicating that nonshared environmental factors (and measurement error) influencing these word reading fluency and print exposure are largely independent.

## Discussion

In this study, we applied bivariate GCTA to examine two predictions derived from twin studies of reading development: that genetic factors partially account for the stability of individual differences in word reading fluency, and that genetic factors contribute to the association between word reading fluency and print exposure. The current results are examined in light of these predictions. We also discuss the correspondence of the GCTA results with the twin study estimates, and we evaluate the merits of GCTA for future studies.

### To What Extent Do Common Genetic Variants Account for Variance in Word Reading Fluency Skills and the Stability of Individual Differences in Word Reading Fluency Skills From Ages 7 to 12?

Our first set of findings concerned the role of genetic factors in the stability of word reading fluency from ages 7 to 12. The phenotypic correlation between TOWRE scores at ages 7 and 12 was 0.69, which is consistent with previous findings that individual differences in early development are relatively stable. GCTA indicated that around one fourth of the variance in TOWRE scores at both ages was due to the aggregate effect of genotyped and imputed SNPs. The GCTA-based heritability was significant at age 12 but not at age 7, although the effect size at each age was similar (29% vs. 21%). Mirroring the phenotypic correlation, the GCTA-based genetic correlation between TOWRE at ages 7 and 12 was 0.69.

These findings suggest that the phenotypic stability of word reading fluency from ages 7 to 12 is largely driven by genetic stability. Developmentally stable genetic effects may reflect genetic influences on a set of core cognitive skills that contribute to individual differences in word reading fluency throughout the middle school years (e.g., working memory). It is also clear that the genetic correlation is not unity, indicating that some genetic variants important during early reading development are less important later on, and vice versa. This pattern may reflect developmental changes in the skills that children draw on when reading words. For example, although the TOWRE is designed to tap both fluency and accuracy, performance is mainly constrained by accuracy in early childhood, whereas fluency becomes a more important factor in determining performance later on (Torgesen et al., 1999). To the extent that genetic factors influencing the processes that support reading accuracy and fluency differ (see, e.g., Hart, Petrill, & Thompson, 2010), the developmental genetic correlation will be attenuated.

### To What Extent Do Common Genetic Variants Account for Variance in Print Exposure and the Correlation Between Print Exposure and Word Reading Fluency Skills?

At a phenotypic level, TOWRE scores at ages 7 and 12 were significantly correlated (~0.45) with ART scores at age 12. This finding is consistent with a large body of evidence demonstrating that higher levels of print exposure are associated with better reading performance (Mol & Bus, 2011). The results from our bivariate GCTA are more difficult to interpret due to the large confidence intervals, but the effect sizes indicate that over 20% of the variance in ART scores at age 12 was due to the aggregate effect of genotyped and imputed SNPs. This finding is consistent with previous twin research showing genetic influences on individual differences in print exposure (e.g., Harlaar et al., 2011; Martin et al., 2009).

Against this backdrop, it is interesting to consider the role of genetic factors in the association between word reading fluency and print exposure, again with the caveat that the confidence intervals from the bivariate GCTA were large. The GCTA-based genetic correlation between 12-year TOWRE and ART scores was substantial and significant (genetic *r* = 0.89). This finding of genetic overlap is consistent with the notion of gene–environment correlations between print exposure and genes that influence variation in reading development, as described in the Introduction. That is, a child at high genetic risk for reading difficulties is likely to show lower print exposure levels compared to the child with a lower genetic risk, possibly because their struggles make reading an unrewarding task that causes the child to avoid opportunities to read (Mol & Bus, 2011). It is unclear from the current results whether there is a genetic path linking *early* individual differences in reading with *later* individual differences in print exposure. The GCTA-based genetic correlation between 7-year TOWRE and 12-year ART scores was moderate but not significant (genetic *r* = 0.33), although the corresponding twin analysis yielded a significant and substantial genetic correlation (genetic *r* = 0.70).

### Correspondence Between GCTA and Twin Estimates

An important feature of this study is that we were able to derive both GCTA- and twin-based estimates of heritability and genetic correlations from the same sample, allowing us to directly compare the results of the GCTA and twin analyses. The GCTA-based heritabilities were much lower than the twin-based heritability. Specifically, the GCTA-based heritability estimates for TOWRE scores were about one third the magnitude of the corresponding twin-based heritabilities, whereas the GCTA-based heritability for ART scores was around two thirds the magnitude of the twin-based heritability. As noted in the Introduction, the missing heritability arises because GCTA only captures the additive effects of common SNPs and causal genetic variants that are in linkage disequilibrium with those SNPs. GCTA is not able to capture genetic variation that is due to rare variants because these variants are not well represented on the DNA array used in this study. Thus, the GCTA-based heritabilities must be regarded as the lower limit of the genetic contributions to word reading fluency and print exposure, reflecting the variance explained by SNPs on the DNA array used in this study. In contrast, the heritability estimated from twin analyses is attributable to DNA sequence variation in any kind (Plomin, Haworth, et al., 2013).

A different picture emerges for the genetic correlations. In the developmental analyses of the TOWRE from ages 7 to 12, the GCTA-based genetic correlation was similar to the twin study estimate (0.71 vs. 0.82). Similarly, in the bivariate analysis of 12-year TOWRE and ART scores, the GCTA-based correlation was substantial and not significantly different from the twin-based correlation (0.89 vs. 0.58). These findings raise a question: Why are estimates for the genetic correlation not significantly different when GCTA-based heritability estimates are only about half the twin heritability estimates? A likely reason is that attenuation of the estimated additive genetic variance in GCTA applies to both the two additive genetic variance components and to the additive genetic covariance in the same proportion (Trzaskowski, Yang, et al., 2013). This will cancel out the bias due to imperfect linkage disequilibrium between causal variants and genotyped SNPs, leaving an unbiased estimate of the genetic correlation.

### Value of GCTA for Studies of Child Development

GCTA does not identify specific genetic variants associated with the measures under investigation, even though it relies on genotyping of genetic variants on DNA arrays. Rather, this method uses random genetic similarity among unrelated individuals to estimate the variance in the phenotype (or the covariance between phenotypes) accounted for by additive genetic factors. It clearly has parallels with twin-based studies, which seek to accomplish a similar goal using the genetic relatedness among twins. Accordingly, it could be asked what value is gained by using GCTA for studies of child development. What can GCTA offer that GWA and twin studies do not?

It is increasingly clear that GWA efforts based on single samples will not succeed in identifying replicable genetic variants, especially if the majority of genetic variants have very small effect sizes (cf. Luciano et al., 2013; Meaburn et al., 2008). As highlighted by the GWA of educational attainment by Rietveld et al. (2013), even “megasamples” of hundreds of thousands of individuals are not a panacea. Due to the tiny effect sizes and the stringent (*p* ≤ 5 × 10^−8^) threshold set for determining genome-wide significance, many real genetic associations are likely to be missed. Nonetheless, studies with genome-wide genotyping data that are under-powered for GWA may still be able to conduct GCTA, which affords the opportunity to answer questions about the aggregate effects of common SNPs on phenotypic variance regardless of whether individual SNPs are significantly associated with the phenotype. Because this method does not depend on reliable estimation of individual SNPs, it should provide unbiased GCTA heritability estimates. It is important to note, however, that GCTA does not obviate the need for large sample size, as is clear from the large confidence intervals in this study of almost 3,000 individuals. The large confidence intervals arise because GCTA estimates depend on extracting a tiny genetic signal (genetic similarity < 2% between each pair of individuals) from the noise of thousands of SNPs on DNA arrays.

It is also the case that identifying specific SNPs remains a valuable and important goal. If we are able to identify SNPs that are robustly associated with reading, these SNPs could be used as aggregated polygenic scores to predict children’s genetic risk for reading difficulties and to investigate developmental, multivariate, and gene–environment issues (Plomin & Simpson, 2013). Moreover, identifying SNPs associated with reading may become more readily achievable with newer technologies. GWA studies are moving increasingly toward sequencing-based approaches that facilitate the analysis of all variants in the entire DNA sequence, including rare as well as common SNPs. This development may overcome the criticism that the incomplete tagging of rarer, potentially causal variants on current DNA microarrays will lead to associations with these variants being missed. In addition, sequencing-based studies are able to detect genetic variants other than SNPs (e.g., insertions, deletions, inversions, and copy number variants) that might be important for individual differences in reading skill, but are often not assayed by DNA arrays. Renewed efforts to identify reading-related SNPs using whole-genome sequencing technologies will also have the advantage of allowing a larger proportion of genetic variance to be captured in GCTA. Thus, GWA and GCTA are likely to have complementary roles in the future, particularly if larger samples can be ascertained (e.g., through multisite consortia) as the costs of genome sequencing fall (Plomin & Simpson, 2013).

A similar balance sheet can be drawn for twin studies, which continue to be widely used in studies of child development. GCTA has several advantages over twin studies. Identifying members of rarer populations such as twins is often difficult and expensive, especially when twin registries are not available. In contrast, aside from the requirement that participants are not from the same familial background, GCTA does not depend on a specific population. This may be particularly important when the phenotype (or environment) of interest is relatively rare and thus not easily studied in selected samples. A further point of note is that assumptions inherent to the classical twin design may not always be tenable. For example, twin analyses assume that MZ and DZ twins share the same trait-relevant shared environments to the same degree (the equal environments assumption), that there is no assortative mating for the trait of interest, and that conclusions based on twin data can be generalized to singletons (Plomin, DeFries, 2013). These assumptions are not required in GCTA.

Twin studies also have several advantages. At the present time, only one or two phenotypes can be examined simultaneously in a GCTA. In contrast, twin analyses readily extend to multivariate questions, as is evident from the longitudinal study of reading across multiple ages (e.g., Christopher et al., 2013; Harlaar et al., 2007a) and analyses of the relationships among print exposure and multiple facets of reading (e.g., Martin et al., 2009). In addition, GCTA provides information about only one parameter: the extent to which variance or covariance is due to SNPs with common additive effects. All other variance is subsumed under a “residual” component. In contrast, phenotypic variance in a twin analysis is decomposed into additive genetic influences and an environmental component, which is subdivided into shared and nonshared environmental influences. More fine-grained analyses of the environmental component are possible with variants of the classical twin design. For example, the longitudinal study of discordant MZ twins may be used to disentangle environmentally mediated risk factors from those with a genetic basis (e.g., Hart, Taylor, & Schatschneider, 2013). Mirroring our evaluation of GCTA versus GWA studies, we suspect that GCTA and twin studies are also likely to be regarded as complementary tools in the analysis of genetic influences on individual differences in child development.

As a roadmap for future work, we suggest two ways in which GCTA could be incorporated more widely into child development research. First, as illustrated by this study, GCTA provides an alternative way to examine some of the research questions traditionally studied in twin samples. In future research, GCTA could be leveraged to address questions about the overlap between reading and other phenotypes (e.g., to what extent do common SNPs account for the comorbidity between reading difficulties and language impairments?). In addition, GCTA may be used to gain purchase on gene–environment interplay in development. In this study, we focused on gene–environment correlation, but Gene × Environment (G × E) interactions could also be investigated in a GCTA framework, where genes are the random effect of all measured SNPs and the environment is a fixed environmental measurement (Vrieze, Iacono, & McGue, 2012). In sufficiently large samples, this method may provide new insights into popular questions such as whether socioeconomic status moderates the magnitude of genetic influences on reading (e.g., Friend, DeFries, & Olson, 2008; Hart, Soden-Hensler, Johnson, Schatschneider, & Taylor, 2013).

Second, GCTA can be applied to selected genomic regions only, permitting estimates of the proportion of variance due to genetic variation at a specific subset of genes. As an example, variation in the liability to schizophrenia has a GCTA-based heritability of around 23%, and most of this variation appears to be attributable to set of almost 2,000 genes involved in central nervous system function (Lee, DeCandia, et al., 2012; see also Cross-Disorder Group of the Psychiatric Genomics Consortium, 2013). For reading disabilities, it may be informative to estimate the proportion of variance accounted for by SNPs in genes implicated in developmental processes such neuronal migration and neurite outgrowth. This research could provide new insights into the primary biological pathways underpinning individual differences in reading skill.

### Limitations and Conclusions

Some limitations temper the conclusions that can be drawn from this study. First, our sample was relatively small for GCTA. As a result, the confidence intervals around our parameter estimates are large. In some cases, this led to some apparently inconsistent findings. For example, the GCTA-based heritability for 12-year ART scores was significantly greater than zero in the bivariate GCTA with 12-year TOWRE scores but not in the analysis with 7-year TOWRE scores, even though the effect sizes were similar. Larger samples would permit more accurate estimation of GCTA-based heritabilities and correlations.

Second, we focused on a limited developmental time span, from ages 7 to 12. The relation between print exposure and reading skill is reciprocal: Individual differences in print exposure both predict and are predicted by individual differences in reading and reading-related skills (Mol & Bus, 2011). Ideally, therefore, we would examine print exposure and word reading fluency concurrently at multiple ages. The focus on only two ages in this study also limits the generalizability of the results. For example, given that the heritability of print exposure may increase developmentally, the extent to which there is genetic overlap between print exposure and reading could vary in older or younger samples. In a similar vein, the extent to which the results generalize to different ethnic groups and societies remains unknown. The importance of including diverse samples in genetic research is highlighted by the Florida Twin Project in reading, which has shown that the etiology of some early literacy skills may differ across economic contexts (Taylor & Schatschneider, 2010; see also Grigorenko & Dozier, 2013).

A third limitation of this study is that we used only a single measure of reading and print exposure. It would be interesting to compare GCTA results across different types of reading skill, particularly given evidence that genetic factors on word reading recognition or fluency are partially distinct from those that influence other reading skills, such as reading comprehension (e.g., Betjemann et al., 2008; Keenan et al., 2006). Likewise, although checklist recognition measures such as the ART are less likely to suffer from social desirability biases compared to self-report questionnaires (Stanovich & West, 1989), these measures are not necessarily a good index for a child’s reading of electronic texts (e.g., blogs, e-mails, websites) or genres such as nonfiction (Spear-Swerling, Brucker, & Alfano, 2010). Thus, it would be useful to have multiple measures of both reading skill and print exposure.

Notwithstanding these limitations, we suggest that the findings from this study have important implications for understanding individual differences in reading. Educational policy discussions on improving reading achievement increasingly emphasize academic benchmarks for evaluating children’s progress. In the United Kingdom, for example, the Department for Education implemented a statutory phonics assessment in 2012 that requires all children to be tested on the TOWRE at the end of their 1st year of formal schooling; children who do not meet the expected average grade level score must receive extra school support (Department for Education, 2013). Although such benchmarks may facilitate earlier identification of children at risk for reading disabilities, they are less valuable if they lead to “teaching to the test.” Furthermore, they ignore the evidence that genetic factors contribute importantly to children’s individual differences in reading. This study has shown that the aggregate additive effect of common SNPs may partly account for the variance in word reading fluency and print exposure, the developmental stability of word reading fluency from ages 7 to 12, and the association between word reading fluency and print exposure. Thus, we need to consider both children’s genetically influenced aptitudes and their genetically influenced “appetites,” such as their proclivity for seeking out experiences that may foster reading development.

How we may better support children’s genetically influenced individual differences in education is still unknown. However, one promising direction is individualized reading instruction. Studies on “Child Characteristic × Instruction interactions” have found that children who begin the school year with below-average vocabulary scores achieve the strongest growth in word reading skills when their school literacy instruction focuses on a combination of formal code-based activities (e.g., focusing explicitly on how to decode words) and child-managed meaning-focused instruction (e.g., independent reading that required the child to actively extract and construct meaning from text). In contrast, children with above-average vocabulary scores at the start of the school year make greater gains in word reading when they spend most of their time engaged in child-managed meaning-focused instruction (Connor, Morrison, & Katch, 2004). Importantly, individualized reading instruction appears to be causally related to variability in children’s reading, and is more effective in improving children’s reading skills than instruction of similar quality that is not individualized (Connor et al., 2013). In the future, it may be possible to combine such research with individual genotyping and GCTA to examine how genetic factors contribute to children’s response to instruction, reading achievement, and propensities to read. The results of this research may lead to further refinements to individualized literacy instruction programs and foster new ways of thinking about effective education that consider nature as well as nurture.

## Figures and Tables

**Table 1:** Sample Ns and Phenotypic Correlations (95% Confidence Intervals in Parentheses)

		Twin analyses			
	GCTA (*n*)	MZ	DZ	*n* pairs	Phenotypic *r*
TOWRE_7_–TOWRE_12_	2,942	535	748	1,283	0.69 (0.66-0.61)
TOWRE_7_–ART_12_	2,931	575	834	1,409	0.46 (0.43-0.49)
TOWRE_12_–ART_12_	2,598	422	644	1,066	0.43 (0.39-0.47)

*Note*. Subscript indicates age (7 or 12 years) at which measure was administered. Phenotypic *r* estimates based on GCTA sample. GCTA = genome-wide complex trait analysis; MZ = monozygotic twins; DZ = dizygotic twins; TOWRE = Test of Word Reading Efficiency; ART = Author Recognition Test.

**Table 2:** Bivariate GCTA and Twin Analysis Results for TOWRE Scores From Ages 7 to 12 (95% Confidence Intervals in Parentheses)

Source of variance	Variance (TOWRE_7_)	Variance (TOWRE_12_)	Correlation (TOWRE_7_–TOWRE_12_)
GCTA			
Genetic	0.21 (0.00-0.46)	0.29 (0.08-0.51)	0.71 (0.30-1.00)
Residual	0.79 (0.54-1.00)	0.71 (0.50-0.92)	0.69 (0.55-0.83)
Twin analysis			
Additive genetic	0.74 (0.66-0.79)	0.68 (0.60-0.76)	0.82 (0.77-0.87)
Shared environment	0.09 (0.03-0.16)	0.07 (0.00-0.15)	0.39 (0.00-1.00)
Nonshared environment	0.17 (0.16-0.20)	0.25 (0.23-0.27)	0.36 (0.31-0.41)

*Note*. Subscript indicates age (7 or 12 years) at which measure was administered. GCTA = genome-wide complex trait analysis; TOWRE = Test of Word Reading Efficiency.

**Table 3:** Bivariate GCTA and Twin Analysis Results for TOWRE-ART Scores at Age 12 (95% Confidence Intervals in Parentheses)

Source of variance	Variance (TOWRE_12_)	Variance (ART_12_)	Correlation (TOWRE_12_–ART_12_)
GCTA			
Genetic	0.22 (0.00-0.44)	0.26 (0.04-0.49)	0.89 (0.32-1.00)
Residual	0.73 (0.51-0.96)	0.78 (0.56-1.00)	0.27 (0.08-0.46)
Twin analysis			
Additive genetic	0.70 (0.62-0.78)	0.39 (0.34-0.54)	0.58 (0.38-0.79)
Shared environment	0.07 (0.00-0.15)	0.32 (0.24-0.40)	0.66 (0.15-1.00)
Nonshared environment	0.23 (0.19-0.26)	0.29 (0.25-0.32)	0.10 (0.00-0.21)

*Note*. GCTA = genome-wide complex trait analysis; TOWRE = Test of Word Reading Efficiency; ART = Author Recognition Test.

**Table 4:** Bivariate GCTA and Twin Analysis Results for TOWRE Age 7 and ART Age 12 Scores (95% Confidence Intervals in Parentheses)

Source of variance	Variance (TOWRE_7_)	Variance (ART_12_)	Correlation (TOWRE_7_–ART_12_)
GCTA			
Genetic	0.28 (0.00-0.46)	0.21 (0.00-0.44)	0.33 (0.00-0.96)
Residual	0.72 (0.44-0.99)	0.79 (0.57-1.00)	0.50 (0.30-0.69)
Twin analysis			
Additive genetic	0.73 (0.66-0.79)	0.27 (0.18-0.35)	0.70 (0.48-1.00)
Shared environment	0.09 (0.03-0.16)	0.42 (0.35-0.48)	0.64 (0.36-0.92)
Nonshared environment	0.18 (0.26-0.20)	0.32 (0.28-0.35)	0.13 (0.07-0.20)

*Note*. Subscript indicates age (7 or 12 years) at which measure was administered. GCTA = genome-wide complex trait analysis; TOWRE = Test of Word Reading Efficiency; ART = Author Recognition Test.
